# The effect of different aqueous solutions ratios of *Ocimum basilicum* utilized in AgNPs synthesis on the inhibition of bacterial growth

**DOI:** 10.1038/s41598-023-31221-7

**Published:** 2023-04-11

**Authors:** Motahher A. Qaeed, Abdulmajeed Hendi, Ahmed S. Obaid, Asad A. Thahe, Abdalghaffar M. Osman, A. Ismail, A. Mindil, Alharthi A. Eid, Faisal Aqlan, Nadir M. A. Osman, Ammar AL-Farga, Saleh M. Al-Maaqar, Ala’eddin A. Saif

**Affiliations:** 1 Physics Department, Faculty of Science, University of Jeddah, Jeddah, Saudi Arabia; 2 Physics Department and IRC Hydrogen and Energy Storage, King Fahd University of Petroleum and Minerals, Dhahran, 31261 Saudi Arabia; 3 Physics Department, College of Science, University of Anbar, Ramadi, Iraq; 4 Department of Medical Physics College of Applied Science, University of Fallujah, Fallujah, Iraq; 5 Chemistry Department and IRC Hydrogen and Energy Storage, King Fahd University of Petroleum and Minerals, Dhahran, 31261 Saudi Arabia; 6 Department of Physics, University of Hafr Al Batin, Hafar Al-Batin, 31991 Saudi Arabia; 7 Chemistry Department, Faculty of Science, University of Jeddah, Jeddah, Saudi Arabia; 8 Chemistry Department, College of Chemicals and Materials, King Fahd University of Petroleum and Minerals, Dhahran, 31261 Saudi Arabia; 9 Department of Biochemistry, Faculty of Science, University of Jeddah, Jeddah, Saudi Arabia; 10 Department of Biology, Faculty of Education, Albaydha University, Albaydha, Yemen

**Keywords:** Biophysics, Physics

## Abstract

This study examined the effect of varying concentrations of *Ocimum basilicum* aqueous extract, which was done via the green synthesis of Silver nanoparticles (AgNPs), on the identification of the most effective concentration for bacteria inhibitory activity. Different concentrations of the aqueous *Ocimum basilicum* extract (0.25, 0.50, 0.75 and 1.00 mM) were used as reducing and stabilizing agent to synthesize AgNPs by means of the reduction method. The crystal structure and morphology of the NPs were characterized UV–Vis spectra, X-ray diffraction (XRD) and scanning electron microscopy (SEM). The antibacterial efficacy of AgNPs was studied against *E. coli* ATCC 35218 using well diffusion, MIC, MBC, and time-kill curve. The dark yellow color of the *Ocimum basilicum* aqueous solution indicates the successful synthesis process of the AgNPs. UV-spectra of the AgNPs display a gradual increase of absorption in sequence with concentration increase of aqueous *Ocimum basilicum* extract solution from 0.25 to 1.00 mM. This, in turn, led to a shift in the wavelength from 488 to 497 nm, along with a change in the nanoparticle size from 52 to 8 nm. The tests also showed a high activity of the particles against bacteria (*E. coli*), ranging between 15.6 and 62.5 µg/ml. Based on AgNPs, it was confirmed that an aqueous *Ocimum basilicum* extract can be used as an effective, reducing and stabilizing agent for the synthesis of different sizes of AgNPs based on the solvent concentration. The AgNPs also proved to be effective in inhibiting and killing bacteria.

## Introduction

Based on research and innovations in different disciplines such as chemistry, engineering, biology and physical sciences, nanotechnology has been inspired by the developments in many sciences of the current era^1^. It is a promising field for a number of researchers because it has multiple applications in life including: bio-sensing materials, vehicles, electronic devices, cosmetics, and antimicrobial applications^2,3^. Although some chemical methods were first used in the synthesis of nanoparticles, the high costs and toxicity, which are associated with the synthesis of nanoparticles, have led to the rise of various issues, resulting in calls for a better alternative that is less toxic, environmentally friendly, and less expensive^4,5^. Researchers have used a number of alternative materials to fabricate nanoparticles such as fungi, algae, bacteria, yeasts, plants and plant extracts^3,6–9^. The green synthesis method is considered the best in terms of many respects, including the availability of plants, medical safety, and absence of health side effects^9^. Phytochemicals in green synthesis can change plant extracts to a stabilizing and reducing agent for the nanoparticles synthesis^10^. The plant used in green synthesis can include onion^11^, banana peel^12^, tamarind^13^, Syzygium Cumini^14^ and *Ocimum basilicum*^15^. *Ocimum basilicum* is a fragrant medicinal plant that is mostly used in traditional medicine^16^. *Ocimum basilicum* plant is also the essential source of compounds such as phenolic acids, sesquiterpenes, phenylpropanoids, anthocyanins and others^17^. These substances can have many functions within the human body. For example, they can act as antioxidants, anticancer and anti-inflammatory, and they also protect ultraviolet rays^18^. The use of silver nanoparticles in many applications in life is essential due to their diverse properties in different products and fields, where their properties are affected mainly by shape and size, as well as interactions with different media^19^. There is also a challenge to reach its best-applied properties. It can be used in a wide range of consumer products. It has an effect on optical properties, conductivity and antibacterial properties. It can be used as biological particles. It is also used to disinfect household, medical and textile devices, where ultra-fine fibers are incorporated into the fabric for high activity. It can be used as an anti-bacterial with cotton fibers containing silver nanoparticles^20,21^.

Several researchers have used *Ocimum basilicum* in green synthesis. For example, Melda Altikatoglu and his team synthesized copper oxide nanoparticles (CuO NPs) by *Ocimum basilicum* plant extract at room temperature, where the size was found to have a range under 70 nm^22^. Further, Hasna Abdul Salam synthesized titanium dioxide nanoparticles TiO_2_-NPs by the green synthesis method using *Ocimum basilicum*, where the nanoparticles were with a size of 50 nm^23^. Highly stable, zinc oxide nanoparticles ZnO NPs were produced by mixing zinc nitrate and *Ocimum basilicum* leaf extract. The synthesized nanoparticles were found to be mostly hexagonal in shape and the average particle size ranged from 9 to 18 nm^24^. They showed that when the concentration of *Ocimum basilicum* leaf extract increases, the size of ZnO nanoparticles can increase. Through the employment of a simple one-step method, Malik and his team synthesized and decorated ZnO NPs with green mediated via *Ocimum basilicum* extract on reducing graphene oxide (RGO) paper. Scanning electron microscopy technology confirmed the spherical shape of the particle size of 31 nm. Moreover, RGO-ZnO inhibited α-amylase and α-glucosidase antidiabetic activities in vitro, and RGO-ZnO NCs showed antibacterial ability with increasing concentration against both Gram-positive (Cocci) and Gram-negative (*E. coli*) strains^25^.

As it is known, the ZCSE6 phage, as a prebiotic, has antibacterial activity against pathogenic bacteria. Hence, Abdallah S. Abdelsattar studied the synergistic effect of zinc oxide nanoparticles (ZnO-NPs) which were synthesized by *Ocimum basilicum* extract. He added the phage to control pathogenic bacteria. The study showed particles that are irregular in shape, with a size of 10–25 nm. Moreover, ZnO-NPs showed biofilm deformability that can be formed by *Staphylococcus sciuri* (*S. sciuri*). It has synergistic antibacterial activity against *Salmonella enterica* (*S. enterica*), when combined with the phage ZCSE6^26^.

AgNPs are one of the simple, cheap and environmentally friendly materials that can be prepared easily by physical^27,28^, biological^29,30^, and chemical methods^31^. Although AgNPs are also used in various fields of application such as wastewater treatment and the textile industry, they are widely used in medical applications^32,33^ such as drug delivery, antimicrobials^34^, larvicideal agent and anti-cancer^35^. Currently the focus is on its use as an anti-virus activity against COVID-19^36,37^.

Abdelsatar et al. used a new method in bacteria that resist the action of antibiotics. By using AgNPs with phage of ZCSE6, AgNPs were made via green synthesis using *Ocimum Basilicum*. Through this study, a synergistic activity was obtained against the growth of microbes, where the researcher found that the MIC and MBC values, which were 6.25 and 12.5 μg/ml, respectively, decreased to 1.5 μg/ml^38^.

Stable AgNps, with a size range of 3–25 nm, were synthesized using aqueous leaf extracts of *Ocimum basilicum* and *Ocimum sanctum*. The AgNps derived from *O. Sanctum* and *O. basilicum,* respectively, showed an inhibitory effect of 89.31 ± 5.32%, and 79.74 ± 9.51%, respectively, against the *bacillus* stearothermophilus α-glucosidase enzyme model^39^. M. Lakshmanan synthesized AgNPs using an *Ocimum basilicum* extract. The as-synthesized silver nanoparticles showed significantly green antimicrobial activity against Gram-positive bacteria (*Staphylococcus aureus*) and Gram-negative bacteria *E. coli*^40^. However, this work aims at controlling the AgNPs size by different percentages of an aqueous solution of *Ocimum basilicum* (0.25, 0.50, 0.75 and 1.00 mM) and investigates the effect of AgNPs size on the inhibition of the action of bacteria.

## Methodology

All data generated or analyzed during this study are included in this published article and its supplementary information files.

### Materials

The materials used in this research are *Ocimum basilicum* leaves, filter papers, Silver nitrates (99.99%) which were purchased from sigma Aldrich, distilled water and glass substrates were purchased from Amazon.sa.

### Basil plant extract preparation

The leaf extracts of *Ocimum basilicum* were obtained from natural populations, which grow in the Riyadh region. The plant was identified by Hosam Elansary and has been vouchered at the College of Food and Agricultural Sciences, King Saud University (Hosam0002213-101)^41^. To prepare the *Ocimum basilicum* extract, the green *Ocimum basilicum* leaves were collected. Our use of plant material and all methods in our research comply with all applicable local, regional, national and international regulations. Next, the leaves were washed to remove the dirt stuck on them, and then left uncovered at room temperature to dry. These leaves were ground with a home blender until they became completely fine powder. After that, 10 g of *Ocimum basilicum* powder were taken with 100 g of distilled water at room temperature in a glass flask and stirred for an hour. The plant extracts were filtered through paper filters, and the extract placed in a dryer at 40 °C until it dried. After that, 1 g of the dry extract was taken and mixed with 100 ml of distilled water. This was done to get a stock concentration of 10,000 ppm. Finally, all solutions were prepared from this stock solution^38^.

### Preparation silver nitrate solution and AgNPs synthesis

Silver nitrate was taken with a weight of 0.179 g and mixed well with 100 g of distilled water to obtain a concentration of 1 mM. Then, 1 ml of *Ocimum basilicum* extract was added to 5 ml of silver nitrate solution (1 Mm). They were stirred together at room temperature until the color of the solution changed, indicating the formation of AgNPs^42^.

### Preparation of glass substrates

The glass substrates were cut as 2.5 × 2.5 cm^2^. Then, they were washed with distilled water several times and dried. After that, they were immersed in acetone of 99% purity to remove surface impurities. Finally, they were placed in the ultrasound machine for 10 min until the sample became ready for drop casting.

### Drop casting

To deposit the solution by the drop-casting method on the sample surface, some steps were done. First, the solution was placed in an ultrasonic device until homogeneity was achieved. Then, the dropper was filled with liquid. After that, the substrate had to be heated to 50 °C. Finally, the solution was distilled onto the substrate homogeneously.

### Characterization

The diffuse reflectance spectra were recorded by using Agilent Technologies, Cary Series UV–Vis-NIR Spectrophotometer for measuring the light absorbance of the prepared materials. An X-ray diffractometer (Rigaku Ultima IV) equipped with a Cu K X-ray source (= 0.15406 nm) was utilized to investigate the structural characteristics. The acquisition 2 theta range was 20–90, and the scan rate was 0.5°/min. The morphology of the prepared samples was examined using field emission scanning microscopy (FESEM, Lyra 3).

## Antibacterial activity of AgNPs synthesized with different aqueous solution ratios of *Ocimum basilicum*

### Bacterial strains and growth

The bio reporter *Escherichia coli* ATCC 35218 used in this study was obtained from Thermo Scientific™ Culti-Loops™. Then, the bacteria were cultured in nutrient broth overnight and stored at − 75 °C in NB broth with 24% glycerol. Next, the bacteria were kept until they were required for use. After that, the bacteria were revived from the glycerol stock for use by culturing in Nutrient-Broth (NB) medium. This was followed by passing bacteria culture to a tube of glass having 10 ml of NB broth a day before the experiment. Finally, they were cultured on a MacConkey agar plate.

### Agar well diffusion assay

Mueller Hinton Agar Media (MHA) was injected with *Escherichia coli* (10^6^ colony-forming units/ml). In every plate of cultivation, seven holes with 6 mm were pierced using a sterile glass pipette. 100 μl of *Ocimum basilicum* treated with AgNPs was put in the holes using different concentrations, and a negative control was the sterilized distilled water (SDW). The plates were brooded for 24 h at 37 °C. The inhibition zone was remarked as the mean ± standard deviation (SD) of triplicate experiments^43^.

### Estimation of Minimum inhibitory concentrations (MIC) of AgNPs

The minimum inhibitory concentrations (MICs) were generally investigated by micro-broth dilution assays via a 96-well Microtiter plate. So, the verification of MICs was done by the micro-diluted broth method, as described by NCCLS which determined the MICs of specimens versus bacteria^44^. The incubation was done for 24 h at 37 °C. After that, 10 μl Resazurin sodium salt dye (R7017 Sigma-Aldrich) was injected into each well^45^. Column 12, which included media as a negative control, confirmed that there was no contamination on the plate during the dish preparation. While column 1 is a positive control, including cultured strain only with the media, columns 2–11 determined the serial dilution of the *Ocimum basilicum* treated by NPs with the media.

### Determination of minimum bactericidal concentrations (MBC)

The minimum bactericidal concentrations (MBC) are the lowest concentration of the AgNPs prepared by *Ocimum basilicum* aqueous solution, which was inoculated with bacteria to be killed. They were spreading 10 μl of the medium from the MIC's microplate contents. This did not show any bacterial growth, even by repeating the inoculation on nutrient agar plates. After that, this was followed by incubation at 37 °C for 24 h. The first well with < 5 colony was considered as negative for growth and reported as the MBC^45^.

### Time-kill curve

For any bacterial strain, the altituter concentration of MIC and Augmentin for AgNPs were determined by Muller Hinton Broth to obtain the time-kill kinetics. The cultures were subjected to aerobic incubation at 37 °C. Afterwards, the AgNPs and the solutions of Augmentin were injected into the well to determine the antibiotic concentrations. The times of 0, 2, 4, 6, 8, 10, 12 and 24 h were followed in the incubation of AgNPs and antibiotic solution into the well. Using sterilized loop, 0.001 µl specimens of the cultures were collected sterilely and planned uniformly on blood agar plate, and subsequently brooded at 37 °C ± 1 for 24 h. Afterwards, the colonies of bacteria in the cultures were counted by means of a plate counter^46^.

## Results and discussion

### UV–Vis spectra of AgNPs

**Figure 1:**
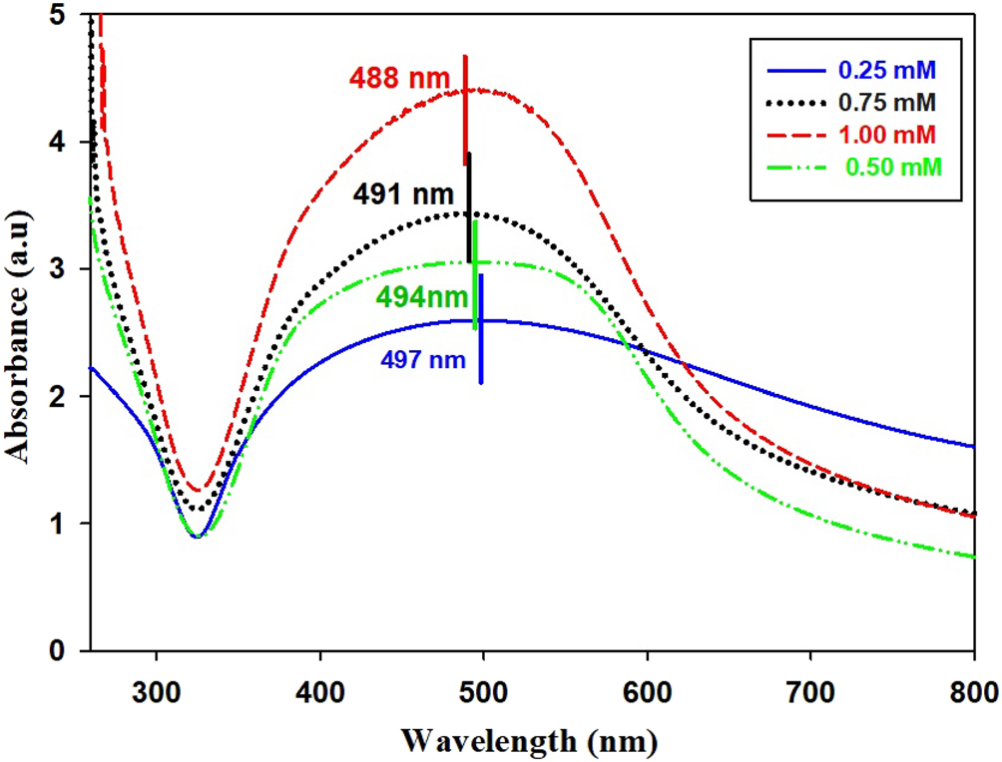
UV–Vis spectra of AgNPs biosynthesized using *Ocimum basilicum* aqueous plant extracts (**a**) 0.25 mM, (**b**) 0.50 mM, (**c**) 0.75 mM, (**d**) 1.00 mM.

**Figure 2:**
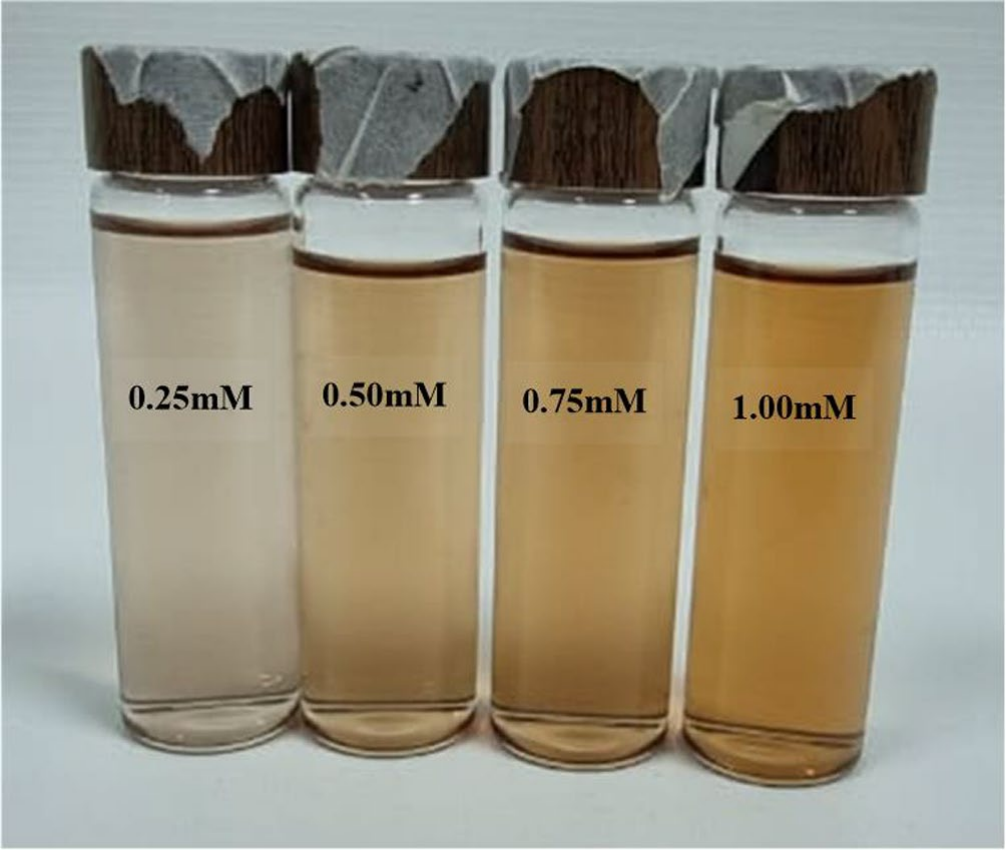
The color gradient of AgNPs liquid from light yellow to dark yellow with different proportions of *Ocimum basilicum* in the synthesis process (0.25, 0.50, 0.75 and 1.00) mM.

The UV absorbed spectra of silver nanoparticles prepared with different ratios (0.25, 0.50. 0.75 and 1.00 mM) of *Ocimum basilicum* extract were recorded. The varied color of the diluted extract from colorless to a light orange color (which can be observed) indicates that the AgNPs formation was obtained as a result of reduction. The absorption band of plasmonic resonance was formed when the light fell on silver nanoparticles containing free electrons, resulting from the mutual vibration between the incident light wave and the electrons. Silver particles and the absorption peaks show the characteristics of plasmonic resonance, indicating a gradual decrease in the absorption from 498 to 488 nm. The peaks in Fig. 1 also indicate the importance of the components of *Ocimum basilicum* leaves, as increasing the concentration of the extract from them leads to an increase in the intensity of absorption^47^. As shown in Fig. 1, the peaks occur between 488 and 497 nm, and there is a shift in these peaks towards increasing the wavelength according to the increase in the particle size associated with a change. The AgNPs solutions colors are shown in Fig. 2. This result corresponds to what was reported in previous studies^48,49^. Thus, the change in the intensity of the absorption came with the change in the concentrations of the *Ocimum basilicum* extract. This could be attributed to the rise in the concentration of the *Ocimum basilicum* extract that led to an increase in the number of compounds in the *Ocimum basilicum* leaves, which were required for reduction from Ag^+^ to Ag^50^.

### XRD of AgNPs analysis

**Figure 3:**
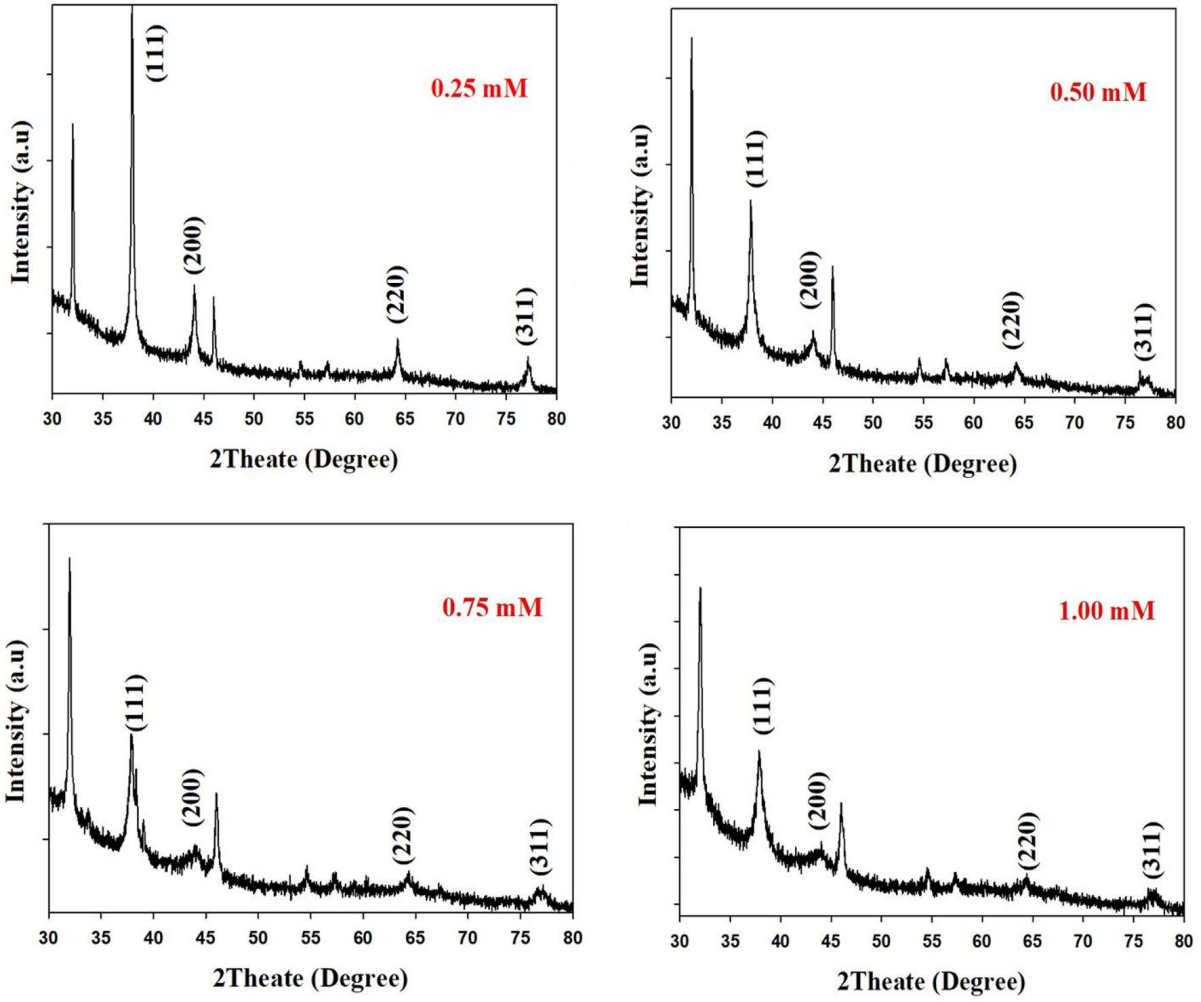
XRD of AgNPs synthesized by different aqueous solutions of *Ocimum basilicum* plant extracts (0.25, 0.50, 0.75 and 1.00) mM.

In this part, AgNPs were synthesized via green synthesis way utilizing *Ocimum basilicum* aqueous solution with different concentrations (0.25, 0.50, 0.75 and 1.00 mM) as a reducing and stabilizing agent. After that, they were studied by XRD to confirm the presence of silver as nanoparticle formation and related structural form. It is clearly shown in Fig. 3 that the peaks that appear at the angles 37.88, 44.07, 64.22 and 77.16 correspond to three levels: (111), (200), (220) and (311), respectively^47^. This corresponds to File No.: 89-3722. This study reports that the AgNPs possess the FCC structure, and the size of these particles can be estimated by Debye–Scherer equation^51^. The estimated average particle size for each sample was determined from XRD results. It was found to be 52.14, 18.48, 52.56 and 8.8 nm for the samples which were prepared with 0.25, 0.50, 0.75 and 1.00 mM of *Ocimum basilicum,* respectively, as shown in Table 1. It is clear that the growth of particles is polycrystalline, and each sample has different peaks. Compared to the rest peaks with low intensities for all samples, the main and high peak was reflected from (111) plane. This can indicate conclusively that the growth at (111) plane was easier than the growth at rest planes^15^. Two unspecified peaks at angles (32.25 and 46.21) were also observed, which might have been caused by the bio-organic compounds that remain attached to the surface of the nanoparticles or perhaps which were caused by the presence of organic compounds available in the leaf *Ocimum basilicum* aqueous solution^52,53^.

**Table 1:** XRD of AgNPs synthesized by different aqueous *Ocimum basilicum* extracts.

Sample	2theat (deg)	FWHM (deg)	D (ang.)	Phase name	AgNPs size (nm)	Average size (nm)
0.25 mM	37.88	0.185	2.37	(1,1,1)	47.4	52.14
	44.07	0.309	2.05	(2,0,0)	29	
	64.22	0.198	1.45	(2,2,0)	49.6	
	76.47	0.126	1.25	(3,1,1)	83.8	
	77.16	0.209	1.24	(3,1,1)	50.9	
0.50 mM	37.85	0.374	2.37	(111)	23.5	18.48
	44.02	0.525	2.06	(2,0,0)	17	
	64.19	0.434	1.45	(2,2,0)	22.6	
	77.17	0.983	1.24	(3,1,1)	10.8	
0.75 mM	37.83	0.502	2.38	(111)	17.5	52.56
	38.34	0.051	2.35	(1,1,1)	172	
	64.21	0.809	1.45	(2,2,0)	12.1	
	76.78	1.252	1.24	(3,1,1)	8.45	
1.00 mM	37.85	0.735	2.38	(111)	11.9	8.8
	44.14	1.362	2.05	(2,0,0)	6.6	
	64.19	1.081	1.45	(2,2,0)	9.1	
	77.00	1.40	1.24	(3,1,1)	7.6	

Jeeva et al. indicated that the crystalline peaks of 32.28°, 46.28°, 54.83°, 67.47° and 76.69°, which appeared in the XRD pattern measurements, were caused by the phytochemical compounds in leaf extracts, where the strongest levels confirmed that silver as a major component in biosynthesis^54^.

### Scanning electron microscopy (SEM) study

**Figure 4:**
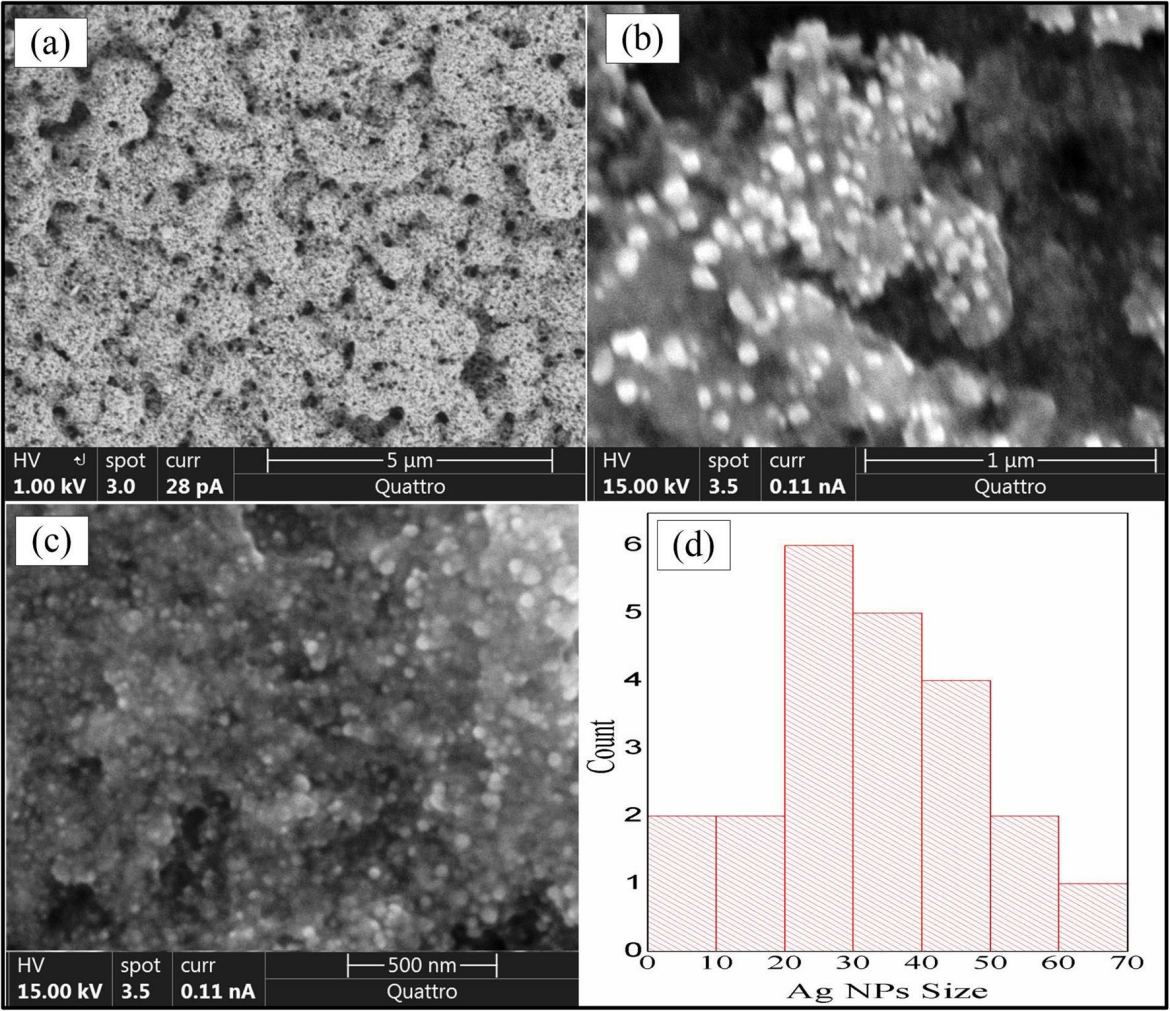
SEM image of AgNPs prepared by 0.50 mM of aqueous *Ocimum basilicum* solution (**a**) 5 µm scale (**b**) 1 µm scale (**c**) 500 nm scale (**d**) distribution of AgNPs sizes.

**Figure 5:**
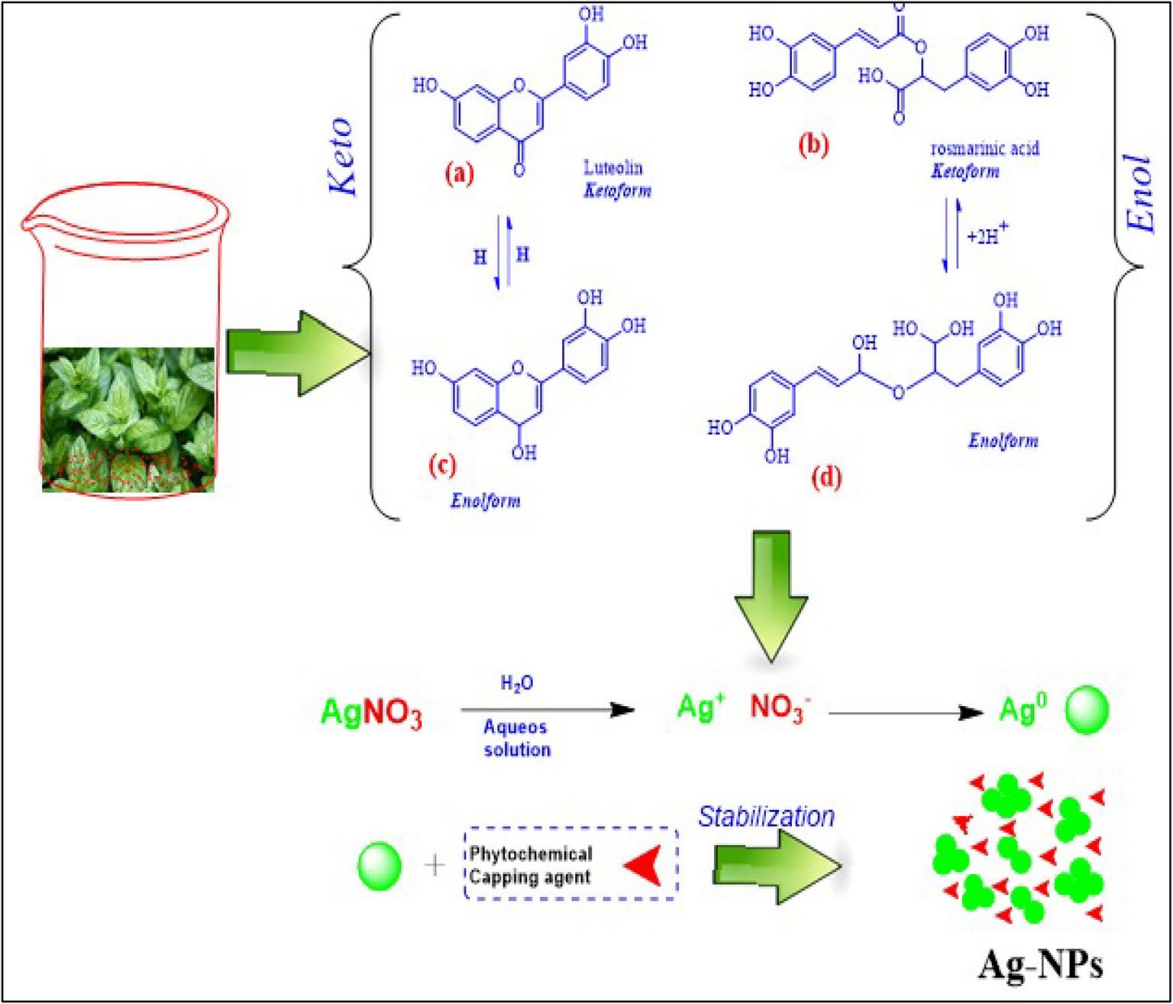
The *Ocimum basilicum* aqueous solution mechanism in synthesis of AgNPs by reduction process to Ag^+^, then, stabilization to AgNPs.

SEM pictures were taken from the sample prepared with 1.00 mM of the *Ocimum basilicum* extract. This clearly indicates the formation of silver nanoparticles AgNPs as spherical shape. According to the presented images, the sample surface included some cavities (voids). There are three different images’ scales in Fig. 5. In these images, the AgNPs appear as separated groups. The average particle size of 20–30 nm was obtained from the image C, as shown in Fig. 4. This can reveal the presence of a strong signal in the UV-spectrum in paragraph 4.1 for this sample due to Surface Plasmon Resonance(SPR)^55,56^.

## Discussion

The *Ocimum basilicum* plant has a high ratio of polyphenols, which plays a key role as an antioxidant. It also contains phenols, flavonoids and other phytochemicals that play a major role in effectively reducing silver salts to nanoparticles. The chemistry of these nanoparticles creates an effective framework for work by circumventing the Particles, making them durable and non-agglomerating. This discovery is critical as it opens new methods for the use of silver nanoparticles in medical applications because it is free of toxicity^15^. Phenolic compounds contain carboxyl and hydroxide groups that have the ability to bind to metals^57^. Plant roots produce a high percentage of phenolic compounds that disrupt the action of ions and do not allow them to bind with other elements through chelation^58^. As flavonoids have a capability to contribute electrons or hydrogen atoms, they are effective as a powerful antioxidant. As shown in Fig. 5a,b, both Rosmarinic acid and luteolin, which are present in the aerial parts of the plant, act as antioxidants and scavenge free radicals. When luteolin is converted to the enol form Fig. 5c,d, this transformation results in the release of the active hydrogen responsible for converting Ag ^+^ to Ag^0^, as shown in Fig. 5.

## Anti-bacterial results of AgNPs

### Antibacterial test using agar diffusion assay

This part of the work aimed at investigating the AgNPs as an antibacterial effect, which were synthesized with different aqueous solution ratios (0.0625, 0.125, 0.25, 0.50, 0.75 and 1.00 mM) of *Ocimum basilicum*. The antibacterial activity of AgNPs was investigated against one species of Gram-negative pathogen *E. coli* ATCC 35218. The results of the well diffusion test, the MIC, MBC, and time-kill kinetics of AgNPs are illustrated separately.

**Figure 6:**
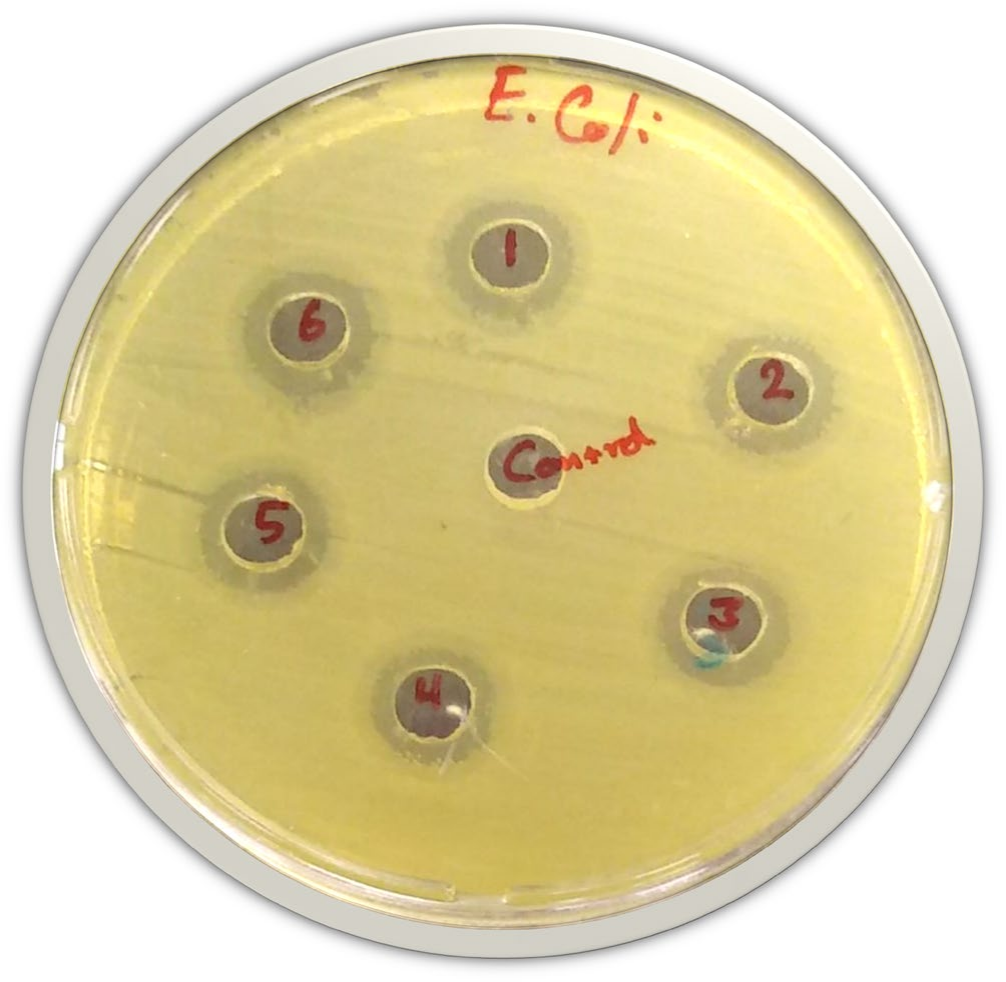
Inhibition zone (mm) of AgNPs synthesized with (1; 0.0625, 2; 0.125, 3; 0.25, 4; 0.50, 5; 0.75 and 6; 1.00 mM)) of aqueous solution Basil by agar Well diffusion method against *Escherichia coli* ATCC 35,218.

Regarding the well diffusion test, the clear zone shown around the AgNPs, which were synthesized by *Ocimum basilicum* aqueous solutions, strongly led to antibacterial activity of the AgNPs, which have the ability to inhibit the growth of pathogenic bacteria. The results of this investigation displayed that the zone inhibition ranged from 10 ± 0.5 mm as the minimum to 12 ± 0.5 mm as the maximum against *Escherichia coli* ATCC 35218. The AgNPs had a variable inhibitory effect, depending upon the concentrations of aqueous solution used in the AgNPs synthesis (0.0625, 0.125, 0.25, 0.50, 0.75 and 1.00 mM) (Fig. 6). The AgNPs had the highest activity (12 ± 0.5 mm zone diameter) against *Escherichia coli* of the AgNPs synthesized with (0.50, 0.75 and 1.00 Mm) aqueous solution *Ocimum basilicum.* They are remarked as sample 1, 5, and 6 in Fig. 6. This can be attributed to the lowest activity (10 mm) against *Escherichia coli* of the AgNPs synthesized with 0.0625 aqueous solution *Ocimum basilicum.* This was remarked as sample 4 in Fig. 6. In this part of the study, there was no significant difference between the concentrations because the ratio of inhibition of bacterial growth was very close, ranging from 10.5 to 12.5 mm. This means that the difference in plant concentrations does not have an effect, compared to the difference of AgNPs synthesized concentration.

### Antibacterial test using MIC and MBC

**Figure 7:**
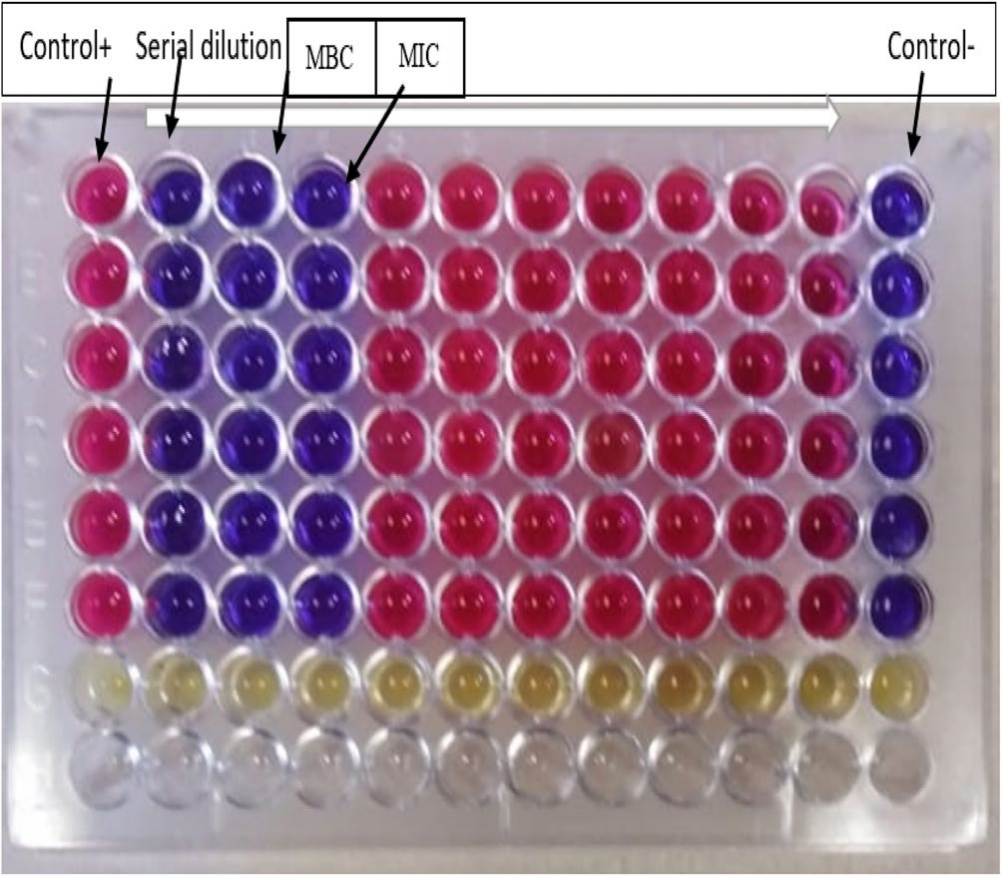
Determination of MIC and MBC AgNPs against pathogenic bacteria (*Escherichia coli*) using Resazurin dye. The rows A–F each represent a samples one to six with pathogenic bacterium (*Escherichia coli*).

The Well diffusion test was done as a preliminary study to explore the antibacterial activity of an antimicrobial agent. However, a more thorough determination in identifying the antibacterial activity of AgNPs synthesized with different aqueous solution ratios (0.0625, 0.125, 0.25, 0.50, 0.75 and 1.00 mM) of *Ocimum basilicum* using the MIC value was required. By serial dilution, the MIC described as the smallest concentration of antibacterial agent was required for inhibiting the growth of bacteria. As shown in Fig. 7, the AgNPs value of MIC against the pathogen (*E. coli*) was 125 µg/ml. MBC is the smallest antibacterial agent concentration that kills the bacteria. In this part of the study, MBC for *E. coli* was 250 µg/ml (refer to Fig. 7). The outcomes of this study are in agreement with those reported in related previous studies^39,41,59^.

### Time-kill kinetics

**Figure 8:**
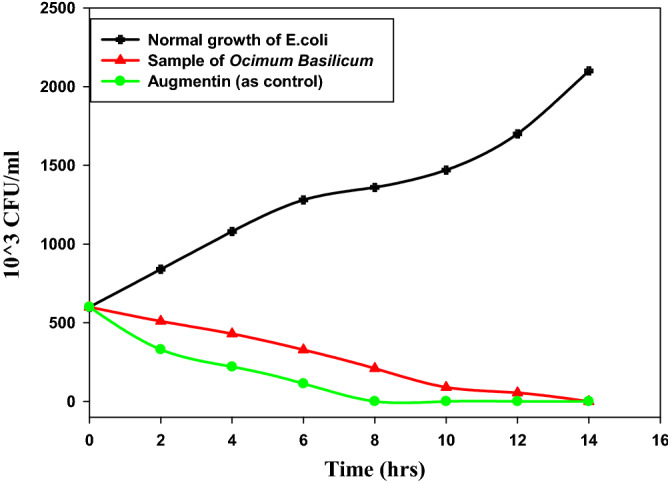
Time–killing curve for *Escherichia coli* ATCC 35218 of *Ocimum basilicum* and Augmentin as control.

Compared to the effect of Augmentin, the time-killing curves of extracting AgNPs revealed less effect, as displayed in Fig. 8. Moreover, based on the MIC test, the values 0.50, 0.75 and 1.00 Mm of *Ocimum basilicum* aqueous solution indicated higher effect, compared to the effect of other samples. After 6–14 h of incubation, all bacteria were completely dead. In the antibacterial absence, the bacterial density for all strains rises rapidly to a peak of 2.1 × 10^6^ bacteria/ml. The ratios of 0.50, 0.75 and 1.00 mM of *Ocimum basilicum* aqueous solution against pathogenic bacteria mentioned above, at the MIC, initially reduced the bacterial numbers. They got killed by AgNPs after incubating for 6 h, whereas the bacterial growth between 14 and 24 h of incubation was zero. This result is consistent with the tests in the previous paragraphs, where 0.50, 0.75 and 1.00 mM *Ocimum basilicum* extract results in smaller AgNPs. This facilitates the process of penetration of bacteria and killing them completely; it is different from the larger particles that suffer a little when penetrating the cell wall of bacteria^60^.

Antimicrobials' long-term effects have commonly been assessed using time-kill curves, which monitor bacterial growth and mortality over a wide range of antibiotic doses. A considerable inhibitory impact against harmful micro-organisms was observed in our investigation (*E. coli*). Based on the findings, the tested micro-organisms died quickly. This might be because AgNPs and *Ocimum basilicum* have different modes of action and because Gram-negative bacteria have different cell wall structures. This decreased the capacity of bacteria to develop resistance against the synthesized NPs in addition to the ongoing stress on the bacteria which were caused by AgNPs at a concentration. The findings of this study are in agreement with what was reported in a previous study^38^.

## Conclusion

In this study, AgNPs were successfully prepared using a less costly, environmentally friendly and less toxic method, which is the green synthesis method. In this method, aqueous extracts of *Ocimum basilicum* used as a reducing and stabilizing agent in different proportions, which acts as a reducing agent. Further, the results revealed that AgNPs appeared with different sizes (8–52 nm). Spherical silver nanoparticles possess a peak absorption in the visible light, and the best concentration is 1.00 mM of *Ocimum basilicum* aqueous extract. This concentration resulted in smaller particles, which helped in penetrating the biological wall of bacteria and killing it.

The antibacterial activity of AgNPs treated with *Ocimum basilicum* against *Escherichia coli* ATCC 35218 was significant. Hence, *Ocimum basilicum* might be a promising candidate for developing AgNPs as an antibacterial agent against pathogenic microorganisms. The use of *Ocimum basilicum* in various fields, such as medical devices and antimicrobial systems, could lead to beneficial discoveries.

## Supplementary Information

Supplementary Information.

## Data Availability

All data generated or analyzed during this study are included in this published article and its supplementary information files.
