# Supplementary material for: The effect of different aqueous solutions ratios of Ocimum basilicum utilized in AgNPs synthesis on the inhibition of bacterial growth
Source: Sci Rep. 2023 Apr 11;13:5866. doi: 10.1038/s41598-023-31221-7 (PMC10088745; doi:10.1038/s41598-023-31221-7)
Supplement: Supplementary file 1 — Supplementary Information. [file 41598_2023_31221_MOESM1_ESM.docx]

**The effect of different aqueous solutions ratios of *Ocimum Basilicum* utilized in AgNPs synthesis on the inhibition of bacterial growth**

Motahher.A.Qaeed^1^, Abdulmajeed Hendi^2^, Ahmed.S. Obaid^3^, AsadA.Thahe^4^, Abdalghaffar M. Osman^5^, A.Ismail^6^, A. Mindil^1^, Alharthi. A. Eid^1^, Faisal Aqlan^7^, Nadir M. A Osman^8^, Ammar AL-Farga^9^ ,Saleh M. Al-Maaqar^10*^, Ala’eddin A. Saif^1^

^1^Physics Department, Faculty of Science, University of Jeddah, Jeddah, Saudi Arabia

^2^Physics Department & IRC Hydrogen and energy storage, King Fahd University of Petroleum and Minerals, Dhahran 31261, Saudi Arabia

^3^Physics Department, College of Science, University of Anbar, Iraq

^4^Department of pharmacy AL-Maarif University College, Iraq

^5^Chemistry Department & IRC Hydrogen and energy storage, King Fahd University of Petroleum and Minerals, Dhahran 31261, Saudi Arabia

^6^Department of Physics, University of Hafr Al Batin, 31991, Saudi Arabia

^7^Chemistry Department, Faculty of Science, University of Jeddah, Jeddah, Saudi Arabia

^8^Chemistry Department, College of Chemicals and Materials, King Fahd University of Petroleum and Minerals, Dhahran 31261, Saudi Arabia

^9^Department of Biochemistry, Faculty of Science, University of Jeddah, Jeddah, Saudi Arabia

^10^Department of Biology, Faculty of Education, Albaydha University, Albaydha, Yemen.

*Corresponding Author: Saleh M. Al-Maaqar ([salehalmagr@gmail.com](mailto:salehalmagr@gmail.com))


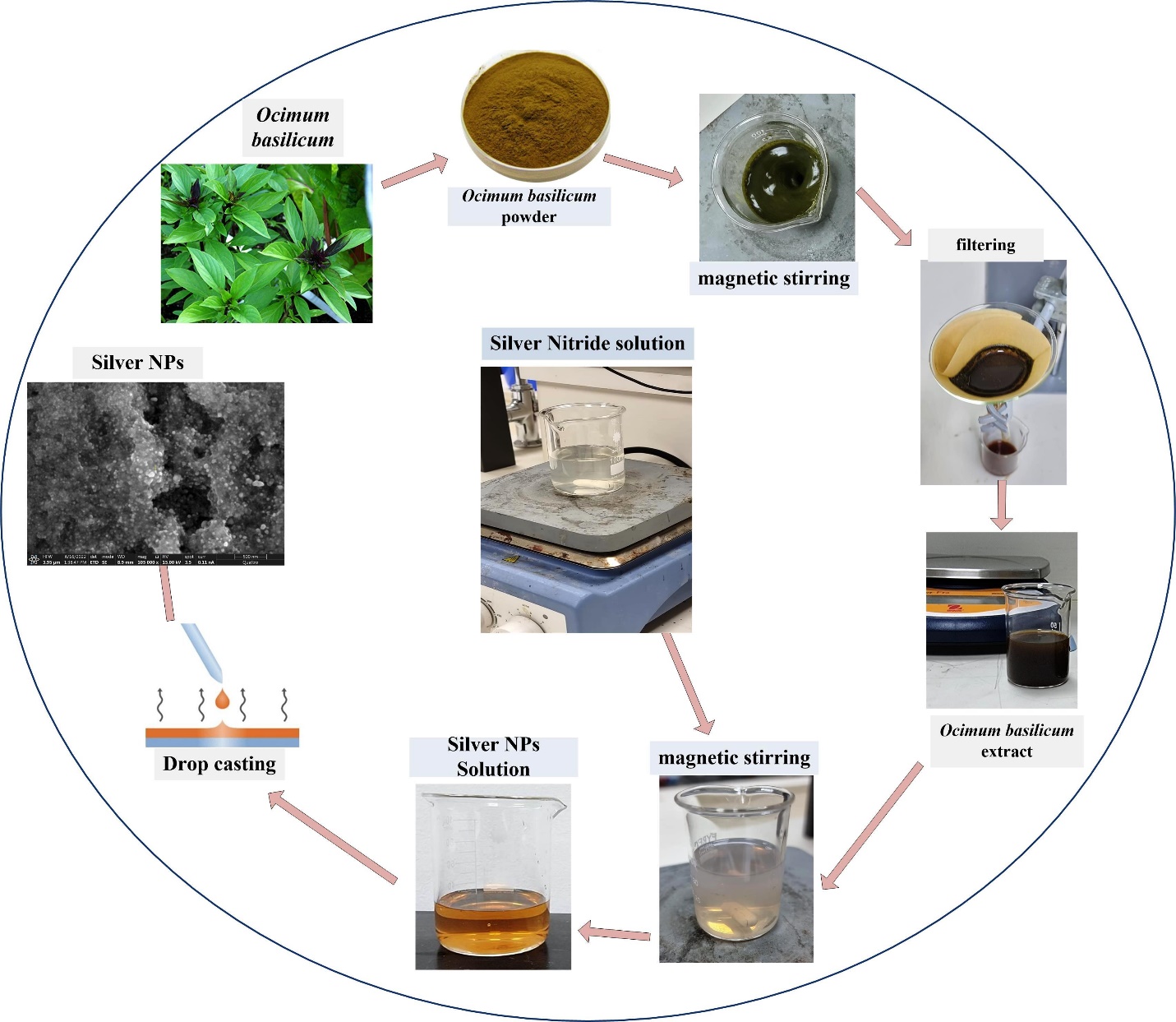


Figure S1 : green synthesis of AgNPs synthesis using *Ocimum Basilicum* plant
